# An Integrative, Multiparametric Approach for the Comprehensive Assessment of Microbial Quality and Pollution in Aquaculture Systems

**DOI:** 10.1007/s00248-021-01731-w

**Published:** 2021-05-04

**Authors:** Loredana Stabili, Marco Di Salvo, Pietro Alifano, Adelfia Talà

**Affiliations:** 1 Department of Biological and Environmental Sciences and Technologies, University of Salento, Lecce, Italy; 2 Water Research Institute of the National Research Council, (IRSA-CNR), Taranto, Italy

**Keywords:** Mariculture system, Microbial pollution indicators, Vibrios diversity, Biolog system, 16S rRNA gene metabarcoding analysis

## Abstract

As the aquaculture sector significantly expanded worldwide in the past decades, the concept of sustainable aquaculture has developed with the challenge of not only maximizing benefits but also minimizing the negative impacts on the environment assuring, at the same time, food security. In this framework, monitoring and improving the microbiological water quality and animal health are a central topic. In the present study, we evaluated the seawater microbiological quality in a mariculture system located in a Mediterranean coastal area (Northern Ionian Sea, Italy). We furnished, for the first time, a microbial inventory based on conventional culture-based methods, integrated with the 16S rRNA gene metabarcoding approach for vibrios identification and diversity analyses, and further implemented with microbial metabolic profiling data obtained from the Biolog EcoPlate system. Microbiological pollution indicators, vibrios diversity, and microbial metabolism were determined in two different times of the year (July and December). All microbial parameters measured in July were markedly increased compared to those measured in December. The presence of potentially pathogenic vibrios is discussed concerning the risk of fish disease and human infections. Thus, the microbial inventory here proposed might represent a new multiparametric approach for the suitable surveillance of the microbial quality in a mariculture system. Consequently, it could be useful for ensuring the safety of both the reared species and the consumers in the light of sustainable, eco-friendly aquaculture management.

## Introduction

The human population is increasing by 83 million each year, as revealed in a FAO report, leading to a daunting challenge of feeding a growing global population, which is expected to reach 8.6 billion by 2030 [1]. This growing global population, indeed, needs a steady supply of high-quality protein. Therefore, to meet this demand for food, aquaculture production, i.e., the farming of aquatic organisms (e.g., fish, mollusks, and crustaceans) and seaweeds must be increased. The need for seafood cannot be gained by capture fisheries alone, due to the overfishing of fish stocks.

Aquaculture offers incontestable advantages, such as the production of good quality and accessible food for the population, the creation of an enormous number of jobs, and the generation of a remarkable budget for the developing countries. Nevertheless, there is an ongoing concern about the environmental risks posed by this economic activity, including water pollution, eutrophication, habitat destruction, biotic depletion, ecological effects, and disease outbreaks that have been and can be caused [2, 3]. In particular, in the marine environment, fish farming causes eutrophication processes since many metabolic by-products, food residuals, fecal matter, and residues of prophylactic and therapeutic inputs are discharged without treatment.

The increased organic matter inputs in the proximity of the fish cages may produce a detrimental effect on seafloor integrity and benthic microbial organisms [4, 5], as well as deterioration of water quality and disease outbreaks [6, 7]. Moreover, conspicuous discharge of pathogen agents as bacteria, viruses, and protozoans from human and animal waste, such as sewage outfalls, accidental spillage and discharge from boats, overload from septic systems during high rainfall events, agricultural practice, and input from native animal species, into the marine environment has become a major alarm for the aquaculture industry [8].

The increase of bacterial pathogen loads in the aquaculture environment leads to two major consequences. The first one is that pathogenic bacteria may produce infections of reared species and, consequently, aquaculture diseases [9]. The second consequence is that the seafood products, severely contaminated by pathogenic bacteria, can pose a severe risk to human health due to human consumption [9].

Most bacteria responsible for disease belong to the normal flora of the water and can cause infection only when the reared species are stressed due to several factors, including unsuitable diet, poor farming techniques, and poor environmental quality. In aquaculture, high mortality rates and skin lesions on fish are due to diseases of microbial origin with subsequent economic losses worldwide in the range of about US$ 6 billion per year [10, 11]. Bacteria, mainly belonging to the genus *Vibrio*, such as *Vibrio ordalii*, *V. harveyi*, *V. vulnificus*, *V. parahaemolyticus*, *V. alginolyticus*, and *V. salmonicida*, have been considered as the etiological agents involved in the most common fish and shellfish disease outbreaks, called vibriosis [12, 13]. Moreover, the accumulation of these microorganisms in the reared animal’s flesh may become a serious threat also to human health.

In addition to vibrios, another important category of microorganisms involved in aquaculture systems management is represented by the microbial pollution indicators. In particular, fecal contamination of the coastal aquatic environments is a major concern. Concentrations of fecal indicator bacteria are commonly used to identify the microbial quality of fish, shellfish, and growing waters [14, 15]. Therefore, based on these considerations, it is evident that microorganisms are of great importance to aquaculture, where they occur naturally or can be unintentionally introduced as contaminants. In the aquaculture environment, microorganisms are involved in nutrients recycling, organic matter degradation, and, occasionally, in infection and death of farmed fishes. Moreover, some microbes may play a role in protecting fish and larvae against disease.

Although the effects of fish farming on sediments and meiofauna have been evaluated in a lot of studies [2, 16, 17], knowledge of the influence of aquaculture practices on the microbial compartment is still scant [18–20], and there is a relatively little knowledge of the effects on the microbial metabolism [21, 22]. As reported in recent studies, the microbial diversity and metabolisms are affected by the local environment and rapidly respond to environmental changes [23, 24]. Therefore, the responses of microbial communities related to environmental changes can be employed as sensitive “sentinels”. Based on this concept, we collectively examined for the first time, a set of microbiological parameters simultaneously measured in a mariculture fish farm located in the Mediterranean Sea, producing the European sea bass *Dicentrarchus labrax* (Linnaeus, 1758) and sea bream *Sparus aurata* (Linnaeus, 1758). In particular, the following microbial parameters were evaluated in two different times (July and December) of the year: total coliforms, fecal coliforms, fecal enterococci, culturable heterotrophic bacteria at 22 °C, culturable heterotrophic bacteria at 37 °C, and culturable vibrios.

Conventional culture-based methods were integrated with the 16S rRNA gene metabarcoding approach for vibrios identification and diversity analysis. Furthermore, in addition to microbial parameters, we evaluated the potential metabolic profiles resulting from the Biolog EcoPlate system, a rapid tool to screen environmental microorganisms by their metabolic fingerprint [25]. Therefore, the paper aims to furnish a microbial inventory and an integrative, multiparametric approach for aquaculture systems management, in order to assess water quality and control the development of microbial infection.

## Material and Methods

### Sample Collection

**Fig. 1:**
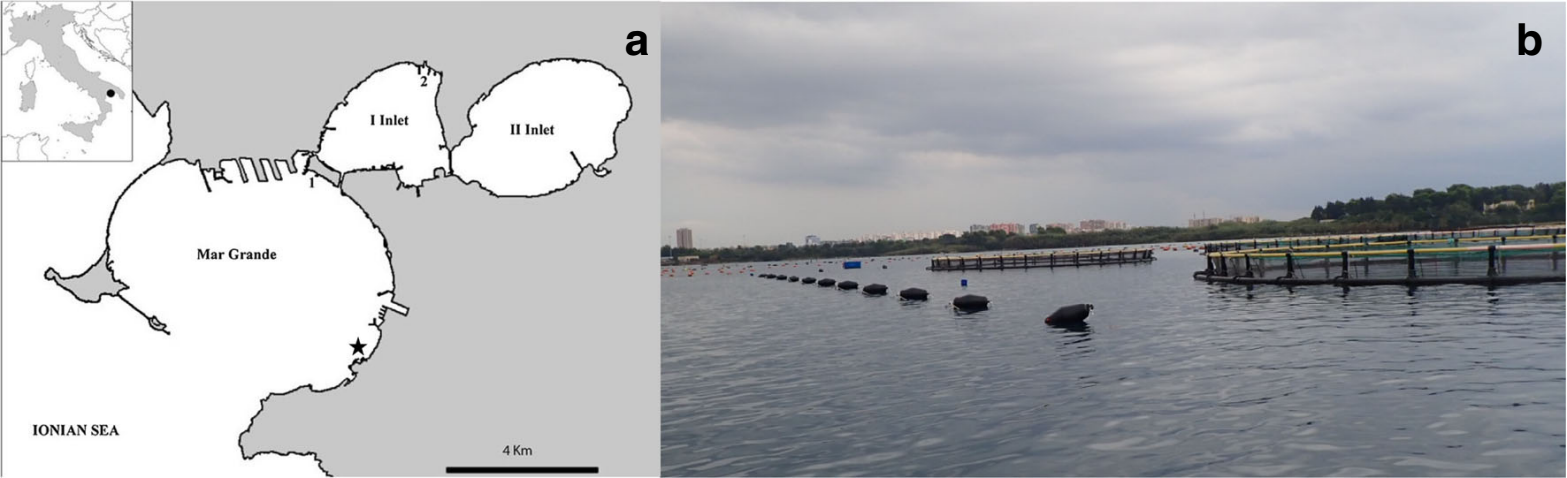
Sampling station in the Mar Grande of Taranto (Northern Ionian Sea, Italy). **a** The geographical location of Maricoltura Margrande fish farm (black star) in the Mar Grande, Italy (full dot in the small box). **b** Mariculture fish farm photograph showing the fish cages with a diameter of about 20 m

In July 2018 and December 2018, seawater samples were collected from a mariculture fish farm (*Maricoltura Margrande*) of 0.06 km^2^, located at Mar Grande of Taranto (Northern Ionian Sea), 600 m away from the coast (Fig. 1). The plant is dedicated to the rearing of sea bass (*Dicentrarchus labrax*) and sea bream (*Sparus aurata*), for a total annual production of about 100 tons. The fish farm consisted of 16 cages with a diameter of about 20 m (circumference of about 60 m) fixed to the bottom through appropriate buoys at a depth ranging from 7 to 8 m (Fig. 1). For each month (July and December), water samples were collected from four replicates sampling points (SP1, SP2, SP3, and SP4) at the periphery of the four fish cages located at the four corners of the farming plant, using 5-L Niskin bottles at a depth of 0.5 m. Niskin bottles were previously washed with 0.1 N HCl and then rinsed with sterilized and filtered (through Millipore 0.2-μm filters) water, as previously reported [26]. Samples were placed on ice and transferred to the laboratory within 4 h for further processing.

### Microbiological Analyses

#### Enumeration of Culturable Bacteria at 22 °C and 37 °C

Traditional plate counting on agar is a classic culture-dependent technique to evaluate bacterial numbers [27]. In the drinking water sector, plate counting on agar is the only method incorporated in the legislation [28]. Within aquaculture, this is not a routine measurement. Despite the scant available data, this approach has been applied in some studies for detecting and/or quantifying bacteria in aquaculture water [29, 30]. Here, in order to evaluate the concentration of culturable heterotrophic marine bacteria at 22 °C, 1 mL of each seawater sample and appropriate decimal dilutions (10^−1^, 10^−2^, 10^−3^, 10^−4^, 10^−5^) were plated in triplicate onto Marine Agar 2216 (MA) [31]. After incubation of the plates seeded on MA in the dark at 22 °C for 7 days, the heterotrophic culturable bacteria were counted, according to the colony-forming units (CFU) method [32]. For total culturable bacteria at 37 °C counting, 1 mL of each seawater sample and appropriate decimal dilutions (10^−1^, 10^−2^, 10^−3^, 10^−4^, 10^−5^) were plated in triplicate onto Plate Count Agar (PCA) [31] and incubated at 37 °C for 24−48 h.

#### Enumeration of Pollution Indicator Bacteria

To assess the microbial water quality in an easy and reproducible way, standard methods (e.g. ISO – the International Organization for Standardization) were followed. In particular, total coliforms and fecal coliforms, as well as fecal enterococci, were determined by the Most Probable Number (MPN) method, using the standard five-tube method of ten-fold dilutions for seawater samples [33]. Coliform bacteria concentration was evaluated by using the miniaturized MPN, in accordance with ISO 9308-3:1998 [34]. Fecal enterococci were measured by using the miniaturized MPN method (incubation at 44 °C for 24–48 h) [35].

#### Enumeration and Isolation of Vibrio Species

Culturable vibrios were enumerated by filtering 1, 5, and 10 mL of each seawater sample on 0.45-μm pore size filters (Millipore). Aseptically, filters were placed onto thiosulphate-citrate-bile-salt-agar (TCBS) plus 2% NaCl, as already reported by [36]. Incubation was performed at 20−25 °C and 35 °C for 2 days. The incubation temperature of 35 °C was selected to estimate the fraction of vibrios potentially pathogenic to humans. The incubation temperature of 20-25 °C was chosen since some *Vibrio* spp., such as *Vibrio anguillarum*, do not grow well at higher temperatures [37]. After incubation, the colonies of presumptive vibrios (yellow or green), grown on TCBS agar, were counted according to the colony-forming unit (CFU) method. Mean values from three replicates were calculated and expressed as CFU/mL.

#### DNA Extraction and Metabarcoding-Based Taxonomic Identification of Cultured Vibrios

All presumptive vibrios colonies grown on TCSB agar, for each replicate sample obtained from the different sites and sampling times, were recovered from the plates and processed for total genomic DNA extraction, according to standard procedures [38]. Extracted DNA was sent to Genomix4life S.R.L. (Baronissi, Salerno, Italy) for 16S rRNA gene metabarcoding and bioinformatics analysis aimed to bacterial identification. Final yield and control quality of extracted DNA were determined using a NanoDrop ND-1000 spectrophotometer (Thermo Scientific, Waltham, MA) and Qubit Fluorometer 1.0 (Invitrogen Co., Carlsbad, CA).

PCR amplifications and 16S rRNA gene metabarcoding sequencing were performed as previously described [39]. Taxonomic assignment of 16S rRNA-targeted amplicon reads was performed using ClassifyReads, a high-performance naïve Bayesian classifier of the Ribosomal Database Project (RDP) [40, 41]. ClassifyReads uses a 32-base word-matching strategy to determine the percentage of shared words between a query and the last available version of the Greengenes taxonomy database (greengenes.secondgenome.com/downloads). In ClassifyReads, the classification confidence is statistically assigned based on the overall accuracy of the classification algorithm at different taxonomic levels (100% for kingdom, 100% for phylum, 100% for class, 99.98% for order, 99.97% for family, 99.65% for genus, 98.24% for species). Reads that did not match a reference sequence were categorized as “Unclassified” [42].

### Biolog EcoPlate Inoculation and Incubation

In the present study, the Biolog EcoPlate system (BIOLOG Inc., Hayward, Calif.) was utilized. It represents a rapid tool to monitor environmental bacteria, evaluating their metabolic fingerprint [25] and has been already profitably employed in several marine environmental studies [43–45]. In this standardized method, the bacterial oxidation of 31 ecologically main carbon substrates, with a redox-sensitive tetrazolium indicator of microbial respiration, is determined [46]. In particular, the metabolized substrates include l-arginine, l-asparagine, l-phenylalanine, l-serine, l-threonine, glycyl-l-glutamic acid, phenylethyl-amine, and putrescine among amino acids; β-methyl-d-glucoside, d-xylose, i-erythritol, d-mannitol, N-acetyl-d-glucosamine, d-cellobiose, glucose-1-phosphate, α-d-lactose, and d,l-α-glycerol phosphate among carbohydrates; pyruvic acid methyl ester, d-galacturonic acid, 2-hydroxy benzoic acid, 4-hydroxy benzoic acid, d-glucosaminic acid, itaconic acid, α-ketobutyric acid, and d-malic acid among carboxylic acids; tween 40, tween 80, cyclodextrin, and glycogen among polymers.

Briefly, from each seawater sample, three replicates were assayed using the Biolog EcoPlates, as previously reported [44]. After the inoculation of each well with 150 μL of the sample, the plates were incubated at 22 °C for 1 week (168 h). The optical density (OD) values were measured at a wavelength of 590 nm with a plate reader (Microplate Reader model 3550; Bio-Rad, Richmond, Calif.). The metabolic activity of the overall microbial community cultivated in Biolog microplates was expressed as average well color development (AWCD) [47]. AWCD values were calculated as absorbance at 590 nm after subtracting control well values [48].

### Statistical Analyses

All statistical analyses were performed using the PAST 4.03 software [49]. The nonparametric two-sample *t* test with Monte Carlo permutation was conducted to evaluate the presence of statistically significant differences between bacterial counts in seawater samples, collected in December and July. Statistical significance was considered at the 0.05 level. A two-sample unpaired Student’s *t* test was conducted to compare the Shannon diversity index for *Vibrio* species in December and July samples. Multivariate Principal Component Analysis (PCA) was performed to evaluate the distribution of culturable bacteria abundance and EcoPlates metabolic parameters at the PAST 4.3 software.

## Results

### Bacterial Enumeration

#### Enumeration of Culturable Bacteria at 22 °C and 37 °C

**Fig. 2:**
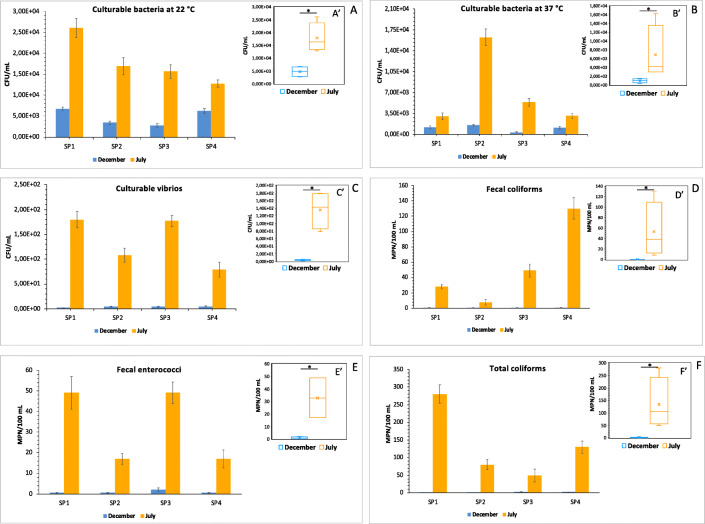
Histograms showing concentrations of culturable bacteria in seawater samples collected in December and July. Bacterial counting is reported as mean values ± S.D. of three replicate plate counts at the four sampling points (SP1, SP2, SP3, SP4). Bacterial counts are expressed as CFU/mL for culturable bacteria at 22 °C (**a**), culturable bacteria at 37 °C (**b**), and culturable vibrios (**c**) and as MPN/100 mL for fecal coliforms (**d**), fecal enterococci (**e**), and total coliforms (**f**). In **a**′–f′, concentration values reported in box-plots for December and July samples correspond to mean values of three replicate plate counts for each sampling point (SP1-4). The asterisk (*) indicates a *p* value

In Figs. 2a and b, bacterial concentrations of culturable bacteria at 22 °C and 37 °C, respectively, are reported as mean values of three replicate plate counts, at the four sampling points (SP1, SP2, SP3, SP4). The mean concentration of culturable bacteria at 22° C in the seawater samples collected in December was 4.8 ± 0.4 × 10^3^ CFU/mL. The counting of these bacteria increased to 1.8 ± 0.2 × 10^4^ CFU/mL in July samples (Fig. 2a′).

Figure 2b′ reports the concentrations of total culturable bacteria at 37 °C in the two sampling times. As already observed for culturable bacteria at 22°C, the highest counting of this bacterial group was observed in July, with a mean value of 6.9 ± 0.7 × 10^3^ CFU/mL. Lower abundances were instead recorded in December, with a mean value of 1.0 ± 0.1 x CFU/mL.

#### Microbial Pollution Indicators

Total coliforms and fecal coliforms showed the highest values in July when 280 ± 26 MPN/100 mL were recorded for total coliforms (Fig. 2f and f′), and 130 ± 14 MPN/100 mL for fecal coliforms (Fig. 2d and d′). In December lower values were observed compared to those recorded in July. The counting of fecal enterococci ranged from 17 ± 3 to 49 ± 6 MPN/100 mL in July, with the mean value of 33 ± 2.7 MPN/100 mL. In December the mean counting of this microbiological parameter was about 1 MPN/100 mL (Fig. 2e and e′).

#### Enumeration and Diversity of Vibrio Species

In Fig. 2c, bacterial concentrations of culturable vibrios are reported as mean values of three replicate plate counts at the four sampling points (SP1, SP2, SP3, SP4). The lowest counting was observed in December, with a mean value of 3.8± 0.9 CFU/mL (Fig. 2c′). By contrast, the vibrios abundance increased in July with a mean value of 1.3 ± 0.1 × 10^2^ CFU/mL.

In this study, we have used a 16S rRNA gene metabarcoding approach to identify, at the species level, cultured vibrios grown on TCSB agar, and to simultaneously estimate their relative abundance. The metabarcoding analysis was performed on each pool of presumptive vibrios colonies recovered from TCSB agar, obtained from the different sites and sampling times. Sequencing of DNA extracted from July and December samples resulted in 749,421 total reads. These reads were quality filtered, and a total of 563,119 reads (ranging from 23,736 to 100,701 per sample) were selected as the most reliable reads for taxonomy assignment. On the whole, a total of 454,884 reads were classified to species level, ranging from 67.19 to 97.33% of total reads per sample. The metabarcoding raw data were refined by calculating the corresponding relative abundance, before using them as input variables for diversity and statistical analyses.

**Fig. 3:**
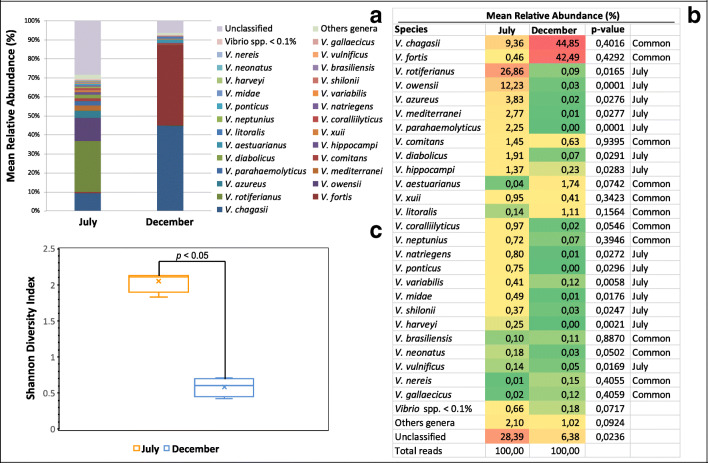
Culturable vibrios diversity. **a** Mean relative percentage contribution for the 26 most abundant (>0.1% within-sample mean relative abundance) vibrios species in July and December samples (*n*=4). **b** Heat map showing the within-sample mean relative abundance of the 26 predominant Vibrio species (>0.1% within-sample mean relative abundance) in July and December pool samples (*n*=4). In **a** and **b**, the <0.1% within-sample mean relative abundances, bacterial genera different from *Vibrio*, and unclassified bacterial species are denoted as “*Vibrio* spp. <0.1%”, “Other genera”, “Unclassified”, respectively. **c** Box plot showing the Shannon diversity index for July (*n*=4) versus December (*n*=4). *p* value displayed is the result of the *t* test between the two groups (*n*=4 per group)

As expected, *Vibrio* was the dominant genus in all samples, other genera have also been detected, with a mean relative abundance of 2.10% and 1.02% for July and December pool samples, respectively. Unclassified reads mean values were significantly more abundant in July samples, indicating a higher contribution of presumptive novel bacterial species and, therefore, greater diversity in July than in December pool samples (28.39% and 6.38% relative abundance, respectively) (Fig. 3a and b).

July and December sampling groups showed markedly different profiles of the most abundant cultured *Vibrio* species (within-sample relative abundance >0.1%, on average). Significant differences (*p*<0.05) were detected in 14 cultured *Vibrio* species (*V. rotiferianus*, *V. owensii*, *V. azureus*, *V. mediterranei*, *V. parahaemolyticus*, *V. diabolicus*, *V. hippocampi*, *V. natriegens*, *V. ponticus*, *V. variabilis*, *V. midae*, *V. shilonii*, *V. harveyi*, *V. vulnificus*), that were predominant in July samples. The relative abundances of the remaining 12 most abundant cultured *Vibrio* species, classified as “common” (*V. chagasii*, *V. fortis*, *V. comitans*, *V. aestuarianus*, *V. xuii*, *V. litoralis*, *V. coralliilyticus*, *V. neptunius*, *V. brasiliensis*, *V. neonatus*, *V. nereis*, *V. gallaecicus*), were statistically comparable between the two sampling groups (*p* > 0.05) (Fig. 3b). Alpha diversity (within-sample diversity) was also investigated using the Shannon diversity index, which accounts for both richness and evenness of the isolated *Vibrio* species. July pool samples had significantly increased Shannon Index (*p* < 0.05), compared to December pool samples (Fig. 3c).

### Bacterial Metabolic Profiles

**Fig. 4:**
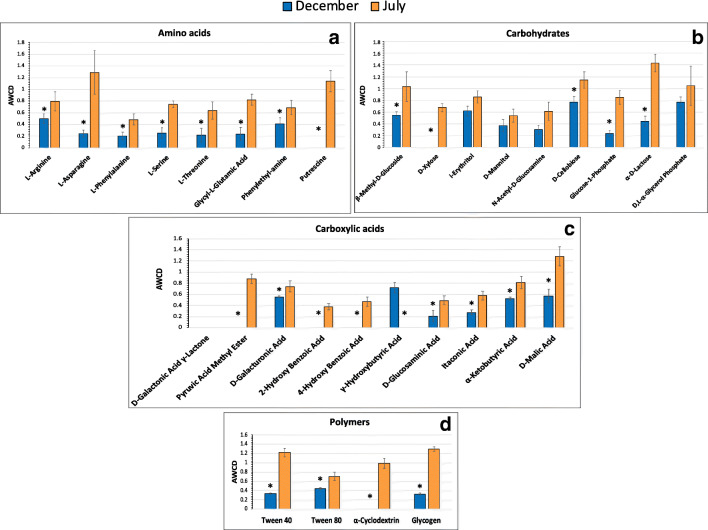
Carbon sources utilization by the microbial communities in the seawater samples collected during the two sampling times. **a** Amino acids, **b** carbohydrates, **c** carboxylic acids, and **d** polymer utilization. Data were shown as mean values of AWCD in all sampling points (SP1-4 December versus SP1-4 July) for each substrate, after 168 h of incubation. Error bars indicate the standard deviation of the mean (*n*=4 per group). Two-sample unpaired Student’s *t* test was used to calculate *p* value (* = *p* < 0.05)

The heterotrophic bacterial communities in the seawater samples, collected during the two sampling times, showed different metabolic patterns based on the 31 carbon substrates included in the Biolog EcoPlate system. Measured in terms of growth over a range of carbon substrates, the highest metabolic activity was recorded for the microbial assemblages associated with seawater collected in July, showing the ability to degrade most of the investigated carbon sources, with the exceptions of two carboxylic acids (γ-hydroxybutyric and d-galactonic γ lactone acids) (Fig. 4a–d). In December the unutilized substrates were putrescine, d-xylose, four carboxylic acids (d-galactonic γ lactone, pyruvic acid methyl ester, 2-hydroxy benzoic and 4-hydroxy benzoic acids) and α-cyclodextrin.

**Fig. 5:**
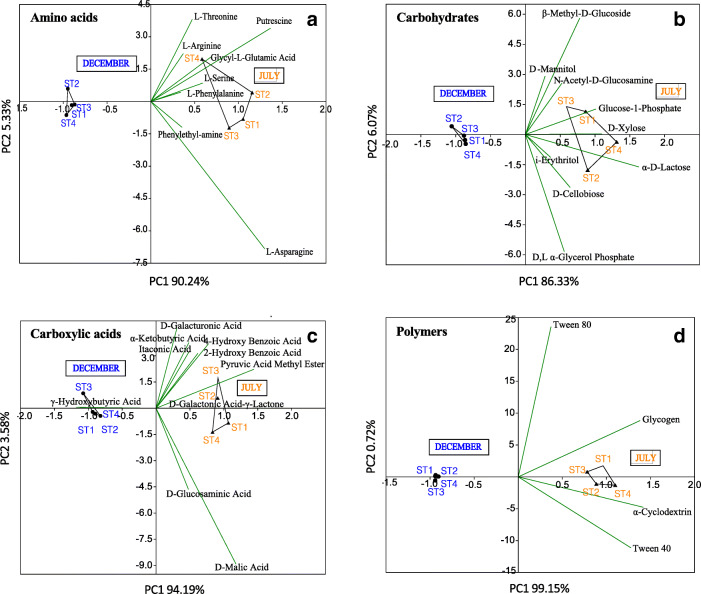
Two-dimensional Principal Component Analysis (PCA) of microbial metabolic parameters for the December and July analyzed samples. **a** Amino acids, **b** carbohydrates, **c** carboxylic acids, and **d** polymer utilization. The input data is a matrix of multivariate data, with sampling groups (*n*=4 per group) in rows and carbon sources in columns. December and July samples are reported by black full dot and black full triangle, respectively. The percentages of the total variance explained by the first and second principal component (P1 and P2, respectively) are also indicated in each plot

PCA multivariate analysis, in Fig. 5a–d, shows overall significant differences in metabolic patterns of the microbial community in the seawater samples collected in the two sampling times. In particular, a clear separation was evidenced in the amino acids, carbohydrates, carboxylic acids, and polymer utilization by the seawater microbial community of July and December (Fig. 5a–d).

### Microbial Inventory Overview

Bacterial enumeration raw data, including culturable heterotrophic bacteria at 22 °C, culturable bacteria at 37 °C, culturable vibrios, total coliforms, fecal coliforms, fecal enterococci plate counts, and EcoPlate AWCD metabolic raw values were logarithmically (base 10) transformed to make variables comparable.

**Fig. 6:**
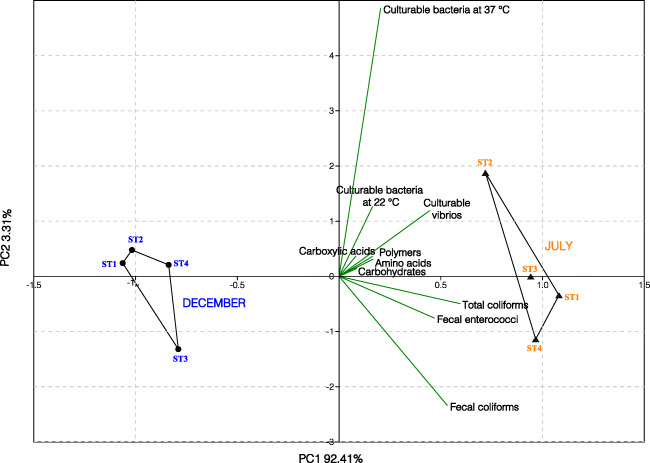
Two-dimensional Principal Component Analysis (PCA) of normalized cultural (culturable bacteria at 22 °C, culturable bacteria at 37 °C, culturable vibrios, total coliforms, fecal coliforms, fecal enterococci) and metabolic (amino acids, carbohydrates, carboxylic acids, polymers) parameters, for the July and December pool samples (*n*=4 for each group). December and July samples are reported by dot and triangle, respectively. The percentages of the total variance explained by the first and second principal component (PC1 and PC2, respectively) are also indicated in the plot

The multivariate PCA of the normalized cultural and metabolic parameters *was performed to summarize and* visualize the overall shape of the monitored variables in mariculture fish farm samples. PCA results are depicted in Fig. 6, where the principal components P1 and P2 explained 92.41% and 3.31% of the variance, respectively. Two main clusters representative of the December and July samples, located on the left and the right side of the P1 = 0 axis, respectively, were visualized in the PCA plot. All the samples SP1-4 collected in July markedly differed from those collected in December for the higher bacterial counting and more versatile metabolic activity, highlighting the relevant impact of seasonality on the fish farm microbial community structure and function.

## Discussion

Microbiological analyses, based on molecular and cultural methods, as well as on the potential metabolic assessment of the seawater microbiota, were performed in a fish farm located in a coastal Mediterranean area (Mar Grande, Northern Sea, Italy). Recently, the aquaculture industry, and particularly mariculture, is expanding worldwide and the challenges for this sector are numerous. These challenges include the development of a more sustainable production ensuring an environmentally sound industry, the availability to consumers of healthy, safe, and good quality products, as well as the promotion of high animal health and welfare standards. Little is known about fish farming impacts in the Mediterranean [20, 50], where rearing of marine species, particularly sea bream (*Sparus aurata*) and sea bass (*Dicentrarchus labrax*), has grown exponentially during the last years. For these reasons microbial water quality control should be regarded as a central management factor/tool to be developed in this sector. In some cases, the most commonly measured parameters in aquaculture facilities are the number of total marine heterotrophs and the number of presumptive *Vibrio*, detected by plate counts [51, 52]. Our study represents a starting point to monitor simultaneously several microbial parameters, also including the potential metabolic profile of the microbial community in a mariculture area of the Mediterranean Sea. From our results, some interesting issues can be inferred.

It is well known that heterotrophic bacteria play an important role in aquaculture, as it has been reported that they account for approximately 80% of the total bacteria, far superior to the number of autotrophic bacteria [53]. Moreover, the number of heterotrophic bacteria in the water is affected by many factors, among which temperature has a great influence [54], and the higher water temperatures in summer are more suitable for bacterial growth. According to these considerations, we recorded higher bacterial counting in July. Values of heterotrophic bacterial counting similar to those observed in the present study have been already recorded in other Mediterranean areas, where mariculture is practiced. In particular, Caruso et al. [55] investigated three Mediterranean sites, where off-shore mariculture was undertaken and found values of 6.5 × 10^2^ CFU m/L (at Capo d’Orlando), 2.85 × 10^3^CFU m/L (at Castellammare Gulf) and 1.95 × 10^3^CFU m/L (at Porto Palo).

Heterotrophic microbial communities in water are also sensitive to changes in nutrient levels [56] deriving from dissolved and particulate substrates provided by aquaculture wastes. High stimulation in the mineralization process may be induced by organic inputs. These may be more or less pronounced depending on the labile or refractory nature of organic matter so that microbial counting and metabolic activities are expected to be enhanced in highly trophic enriched environments, such as intensive aquaculture farms [18]. According to these considerations, we evaluated the metabolic profiles of the microbial assemblages present in the seawater samples.

Our results showed that the aquaculture system was characterized by a microbial community particularly active in organic matter utilization. It is well known that dissolved organic matter (DOM) in the marine environment is a mixture of organic compounds and comprises both low and high-molecular weight solutes, including organic acids, carbohydrates, amino acids, proteins, nucleic acids, lipids, and polymers. Our data showed that most of the amino acids, carbohydrates, carboxylic acids, and several polymers present in the Biolog system were degraded by the microbial community living in the studied area. This result suggests that DOM inputs, coming from sewage wastes, operated as a trophic source, stimulating microbial metabolism and growth.

Moreover, according to the higher bacterial counting recorded in July, the microbial community associated with the seawater in July showed also more versatile substrates utilization patterns compared to the December microbiota. In this regard, we noticed that l-asparagine was the C source more degraded in July. l-asparagine is a neutral amino acid with polar side chains. l-asparaginase is among the relevant enzymes that can be obtained from marine sources [57]. This amidohydrolase is active on l-asparagine, producing l-aspartate and ammonia, and has also some l-glutaminase activity [58–60]. In this framework, it is noteworthy that several *Vibrio* species possess l-asparaginase activity [61].

Fluctuations in the abundance of vibrios, with higher concentrations in July compared to December, reflected the trend recorded in the distribution of microbial metabolism, as well as the counting of total marine heterotrophic bacteria, and were consistent with the ecology of these bacteria, whose growth is increased by warm temperature and high nutrient availability [62]. In general, in aquaculture systems, it has been found that, of all the environmental factors, the temperature is the main driving factor of the bacterial community [63, 64]. Thus, aquaculture outbreaks of aquatic animal diseases are often seasonal, and these outbreaks could be closely related to seasonal changes in microbial populations [65]. Bacteria belonging to the genus *Vibrio* are of particular concern, as they constitute a considerable part of marine halophilic bacterial populations, are strongly thermo-dependent and are often associated with human and marine animals’ diseases, called vibriosis.

Although vibriosis is one of the largest constraints on aquaculture production and one of the major bacterial diseases observed in cultured marine fish worldwide, an epidemic trait of the *Vibrio* in a Mediterranean mariculture farm is still lacking. Our study is an attempt in this direction. In the examined area we evidenced a core of recurrent vibrios, that do not comprise human pathogens, but some of them represent a serious threat for reared organisms [66–73].

Most of the core recurrent vibrios recorded in our study belong to new species in the genus *Vibrio* described, in the last decade, associated with marine environments and aquatic eukaryotic organisms. To mention some examples, *V. litoralis* has been firstly isolated from seawater in a Yellow Sea tidal flat in Korea [74]; *V. comitans* was isolated for the first time from the gut of the abalones *Haliotis discus discus*, *H. gigantea*, *H. madaka* and *H. rufescens* [75]. *Vibrio brasiliensis* and *V. xuii* have been detected in the marine aquaculture environment (bivalves, fish, rotifers, and shrimps) [76].

In July, vibrios diversity increased significantly. Among the identified species, the presence of *V. rotiferianus*, *V. parahaemolyticus*, *V. harveyi* and *V. vulnificus* is noteworthy. In particular, *V. vulnificus* is an opportunistic human pathogen, commonly found in warm coastal waters and in the Mediterranean Sea [77]. It can cause severe gastroenteritis from the consumption of raw seafood, as well as wound infections and necrotizing fasciitis, thus posing a health risk to both seafood consumers and fishermen [78]. Infection due to this bacterial species in fish mainly consists of penetration of bacterium to the host tissue, mainly by the chemotactic activity, followed by deployment of the iron sequestering system and eventually damages the fish through extracellular products i.e., haemolysin and protease [11]. *Vibrio parahaemolyticus*, a seafood borne pathogen coming from the marine environment [79], is responsible for gastroenteritis, sepsis, or wound infection in humans that are infected by eating contaminated raw seafood or wound-related infections [80]. This *Vibrio* species is an aquatic zoonotic agent and can infect also aquatic animals causing vibriosis in several species of fish, shellfish, and other aquatic animals [81]. In aquaculture reared animals, mainly shrimp, *V. parahaemolyticus* causes Acute Hepatopancreatic Necrosis Disease (AHPND) or Early Mortality Syndrome (EMS) with economic losses in this sector worldwide [82].

Among the identified species, vibrios of the Vibrio Harveyi clade were present. These are major pathogens of many aquatic organisms, including vertebrates and invertebrates [83]. In particular, *V. rotiferianus*, a marine pathogen causing disease in various aquatic organisms, was originally isolated from cultures of the rotifer *Brachionus plicatilis* which are important nutrients for fish and crustaceans in aquaculture industries [84]. With the expansion of aquaculture, *V. harveyi* has been identified as a serious source of disease responsible for large-scale mass mortality in shrimp farms [85] and is also pathogenic to oysters, fish, seahorses, and lobsters [86]. The presence of *V. rotiferianus*, *V. parahemolyticus*, *V*. *harveyi*, and *V. vulnificus* in July, in the analyzed seawater samples, led to conclude that in this period of the year marine life is particularly vulnerable to vibrios diseases. This is in agreement with the features of vibrios, which are strongly thermo-dependent and potentially present in a viable but nonculturable (VBNC) state in cold months [17, 87, 88]. Several studies, indeed, have demonstrated that the occurrence of *Vibrio* bacteria in the NW Mediterranean Sea is climate-linked and can vary greatly under the influence of temperature [17, 87, 88].

It must be pointed out here that, as it is well known, the examination of intragenus heterogeneity could be limited by the low species-level discriminatory power, particularly among highly genetically related *Vibrio* species [63, 89]. However, 16S rRNA gene metabarcoding sequencing has been and still is used to characterize *Vibrio* community structure [90, 91]. On account of these considerations, many efforts are currently underway to improve the taxonomic resolution of *Vibrio* diversity, and increase the proportion of sequences that can be unambiguously assigned to *Vibrio* species [92–94].

According to the increase of vibrios diversity, all the other monitored microbiological parameters increased in July. It is well known that the enterococci constitute an indicator for older fecal contamination since they survive better in the environment [95]. Both coliforms and enterococci are frequently used as indicator organisms of fecal contamination of potable and recreational water, as well as food. By contrast, enterococci are not mentioned in the current EU-regulations on the assessment of farming localities for bivalve cultivation. On account of these considerations, we enlarged the spectrum of the microbiological analyses and evaluated both coliforms and fecal enterococci. All the recorded values of microbial pollution indicators were lower than the legal limits of the national and European law, concerning the mussel culture.

Last but not least, of particular interest are the results deriving from the *multivariate statistical data analysis by PCA*. In this analysis, the pattern arising from the structural and metabolic parameters examined in the two sampling times showed a clear-cut separation between the samples collected in July (characterized mainly by high values of all the examined parameters), and the samples collected in December (characterized mainly by low recorded values). This clear separation allows one-shot microbial multicomponent detection that could be useful to the farmers working in the Mediterranean area to realize a more eco-friendly and sustainable aquaculture approach.

An integrated management perspective should indicate in which period of the year (July and summer) the attention must be pointed to prevent possible disease in the aquaculture farms when the microbial load increases. On account of our results, we strongly suggest improving the microbial surveillance of farming waters in Mediterranean areas in summer. Thus, from this preliminary study, some interesting conclusions can be inferred in the field of environmental concerns. Indeed, the observed responses of the microbial community, in the two sampling times, indicate that the here selected microbial inventory represents a “good indicator” to furnish a clear scenario of the environment, where the mariculture is practiced. Therefore, it could be suggested as an integrative, multiparametric approach for a better surveillance of the microbial quality of farming waters. This is particularly important taking into consideration that, at present, as regards the quality of waters the only regulatory and legal constraints in aquaculture policies are concerning shellfish. Up to now, the EU’s water policy is regulated under two instruments: the Water Framework Directive, covering inland and coastal waters; and the Marine Strategy Framework Directive 2008/56/ EC, covering marine waters. This is due mainly to a frequent lack of recognition for aquaculture as an equal user of water resources as compared with other users, such as fisheries or tourism. However, a recent paper by Caruso et al. [21] recommended that the structural or functional prokaryotic variables (biodiversity, abundance, and metabolism) should be included in future implementations of the Marine Strategy Framework. In this scenario, our paper represents a first preliminary attempt toward this direction.

## Conclusions

At present, studies on the microbial communities developing in mariculture areas are still limited. Our paper provides a first insight into the attempt to furnish a set of microbiological parameters, based on culture and molecular methods, useful to assess the water quality in a coastal area of the Mediterranean, where mariculture (*Dicentrarcus labrax* and *Sparus aurata*) is practiced. In the rearing, environmental bacterial counting, versatile metabolic pathways for the microbial community, and a broad vibrios diversity were recorded, with higher values especially in July. Within the vibrios community, we identified a core set of *Vibrio* species, critical to define the health status of the farming environment. Vibrios diversity rose significantly in July and among the identified species the presence of *V. rotiferianus*, *V. parahemolyticus*, *V. harveyi*, *and V. vulnificus* is important for the human and fish disease implications. The analytical approach here suggested could implement the array of the existing strategies for microbial contamination evaluation. Aside from their role for human health risk assessment, the insertion of microbial variables as susceptible indicators of overall ecosystem health status has also been suggested by recent directives on the sustainable management and safeguard of marine and coastal ecosystems. Thus, the here furnished microbial inventory could be suggested as a multiparametric approach for water quality monitoring and the development of more sustainable aquaculture since a clear separation of the measured parameters (structural and functional) was evidenced in the two considered sampling times.

## Data Availability

Not applicable
